# The Trace Conditional Learning of the Noxious Stimulus in UWS Patients and Its Prognostic Value in a GSR and HRV Entropy Study

**DOI:** 10.3389/fnhum.2020.00097

**Published:** 2020-04-09

**Authors:** Daniela Cortese, Francesco Riganello, Francesco Arcuri, Lucia Lucca, Paolo Tonin, Caroline Schnakers, Steven Laureys

**Affiliations:** ^1^Research in Advanced NeuroRehabilitation, Istituto Sant'Anna, Crotone, Italy; ^2^Coma Science Group, GIGA-Consciousness, University & Hospital of Liege, Liege, Belgium; ^3^Neurosurgery Department, University of California, Los Angeles, Los Angeles, CA, United States; ^4^Research Institute, Casa Colina Hospital and Centers of Healthcare, Pomona, CA, United States

**Keywords:** pain, unresponsive wakefulness syndrome, disorders of consciousness, trace conditioning, HRV (heart rate variability), entropy, conditional learning, Galvanic Skin Response (GSR)

## Abstract

The assessment of the consciousness level of Unresponsive Wakefulness Syndrome (UWS) patients often depends on a subjective interpretation of the observed spontaneous and volitional behavior. To date, the misdiagnosis level is around 30%. The aim of this study was to observe the behavior of UWS patients, during the administration of noxious stimulation by a Trace Conditioning protocol, assessed by the Galvanic Skin Response (GSR) and Heart Rate Variability (HRV) entropy. We recruited 13 Healthy Control (HC) and 30 UWS patients at 31 ± 9 days from the acute event evaluated by Coma Recovery Scale–Revised (CRS-R) and Nociception Coma Scale (NCS). Two different stimuli [musical stimulus (MUS) and nociceptive stimulus (NOC)], preceded, respectively by two different tones, were administered following the sequences (A) MUS1 – NOC1 – MUS2 – MUS3 – NOC2 – MUS4 – NOC3 – NOC^*^, and (B) MUS1^*^, NOC1^*^, NOC2^*^, MUS2^*^, NOC3^*^, MUS3^*^, NOC4^*^, MUS4^*^. All the (^*^) indicate the only tones administration. CRS-R and NCS assessments were repeated for three consecutive weeks. MUS4, NOC3, and NOC^*^ were compared for GSR wave peak magnitude, time to reach the peak, and time of wave's decay by Wilcoxon's test to assess the Conditioned Response (CR). The Sample Entropy (SampEn) was recorded in baseline and both sequences. Machine Learning approach was used to identify a rule to discriminate the CR. The GSR magnitude of CR was higher comparing music stimulus (*p* < 0.0001) and CR extinction (*p* < 0.002) in nine patients and in HC. Patients with CR showed a higher SampEn in sequence A compared to patients without CR. Within the third and fourth weeks from protocol administration, eight of the nine patients (88.9%) evolved into MCS. The Machine-learning showed a high performance to differentiate presence/absence of CR (≥95%). The possibility to observe the CR to the noxious stimulus, by means of the GSR and SampEn, can represent a potential method to reduce the misdiagnosis in UWS patients.

## Introduction

Disorders of Consciousness (DOC) are a spectrum of pathologies affecting one's ability to interact with the external world. They are increasingly becoming a worldwide health concern, whether due to a traumatic (Meaney et al., 2014; Roebuck-Spencer and Cernich, 2014) or non-traumatic cause (Gitler et al., 2017; Erkkinen et al., 2018), with its share of ethically challenging questions (Fins, 2005; Demertzi et al., 2011; Riganello et al., 2016), including life and death decisions. Indeed, differential diagnosis of the clinical entities of disorders of consciousness raises important ethical and medical issues, including pain treatment and end-of-life decisions.

Despite a unifying term being used, these disorders do in fact cover a broad population of very heterogeneous pathologies with diverse etiologies, injuries, and outcomes. This heterogeneity can make them hard to distinguish in the clinical practice (Fins, 2005), leading the examiners to a possible misdiagnosis, that is reported around 30% (Andrews et al., 1996; Bosco et al., 2010; Peterson et al., 2015; van Erp et al., 2015).

Unresponsive Wakefulness Syndrome (UWS) (Laureys et al., 2010) and Minimally Conscious State (MCS) (Giacino et al., 2002) are two of the possible conditions following an acquired brain injury. The MCS patients exhibit minimal but discernible signs of non-reflex behaviors which occur reproducibly (yet inconsistently) as a response to visual, auditory, tactile, or noxious stimuli; conversely, UWS (Laureys et al., 2010) condition is characterized by a spontaneous opening of the eyes and no sign of consciousness, but reflexive responses to external stimuli (Jennett, 2002; Dolce et al., 2010).

The clinical assessment of UWS condition is based on clinical consensus and behavioral scales, such as the Coma Recovery Scale-Revised (CRS-R) (Giacino et al., 2004). The difficulty in the assessment of the consciousness level of UWS patients often depends on a subjective interpretation of the observed spontaneous and volitional behavior (Cortese et al., 2014). In the absence of speech, the motor response is the only observable behavior. The behavioral response to nociceptive stimulation, relying on a wide brain network linked to the consciousness, can help the examiner to evaluate the change in the level of consciousness (Schnakers et al., 2010; Riganello et al., 2014; Chatelle and Laureys, 2015). However, in the absence of any possible cognitive output, it represents a strong challenge in detecting the conscious perception of the pain in UWS patients (Chatelle and Thibaut, 2014; Naro et al., 2015; Schnakers and Zasler, 2015; Garcia-Larrea and Bastuji, 2018).

It has been observed that nociceptive stimuli elicit the activation of an extensive cortical network that includes somatosensory, insular, cingulate, frontal, and parietal areas (Pain Matrix) (Coghill et al., 2003; Iannetti and Mouraux, 2010; Chatelle et al., 2014).

The nociceptive stimulation activates the nociceptors, and via the spinothalamic tract, the information reaches the thalamus and the cortex, where the midbrain and thalamus are thought to be involved in the modulation of reflex responses to nociceptive stimuli (Loeser and Treede, 2008). The secondary somatosensory (S2) cortex, with the posterior insula (lateral network), are also involved, taking part in the sensory–discriminative features of pain processing (Ploner et al., 2002; Lockwood et al., 2013).

However, the concept of “Pain Matrix” is often used to explain the generation of the conscious experience of pain. Pain experience is defined as “an unpleasant sensory or emotional experience that is associated with actual or potential tissue damage, or which can be described in terms of such damage.” (Loeser and Treede, 2008). Given the subjective nature of pain, and the impossibility for a UWS patient to discuss it, it is not possible to report his/her response to a nociceptive stimulus as pain sensation [nociception refers to the perception -conscious or not- of nociceptive stimuli (Loeser and Treede, 2008)], although it is also not possible to exclude it (Naro et al., 2015; Calabrò et al., 2017).

Pioneering studies attempted to quantify the nociception in patients with DOC (Schnakers et al., 2010). The Nociception Coma Scale (NCS) (Schnakers et al., 2010; Chatelle et al., 2014; Riganello et al., 2014), a behavioral tool, was developed specifically for DOC patients and measures eye-opening, breathing, and grimace-like or crying-like behaviors (Schnakers and Zasler, 2007).

In clinical practice, electroencephalography (EEG) recordings (Gantner et al., 2012; Fernández-Espejo and Owen, 2013) and neuroimaging approaches (Schiff et al., 2005; Turner-Stokes et al., 2012) have been proposed, and are often used, as complementary tools to help in assessment, diagnosis, prognosis, and decision making in DOC patients (Cruse et al., 2011; Di Perri et al., 2014; Demertzi et al., 2015). Both fMRI and EEG recordings are hardly doable, because of movement artifacts (Havsteen et al., 2017; Jiang et al., 2019) or, in the case of EEG recordings, because of the presence of craniotomy (Reis et al., 2014).

Several studies investigated the extent to which the pain matrix responded to nociceptive stimuli in patients with DOC by a laser-evoked EEG response (LEPs) (Tommaso et al., 2013; de Tommaso et al., 2015; Naro et al., 2016), suggesting the presence of covert pain processing also in subjects with low Nociception Coma Scale-Revised (NCS-R) scores.

Neuroimaging studies suggest that UWS patients could maintain primary and/or more complex cortical activation in response to noxious stimuli, but this would occur as an isolated and disconnected phenomenon preventing a conscious perception of pain (Laureys et al., 2002; Kassubek et al., 2003; Kotchoubey et al., 2013; Markl et al., 2013). However, cortical activation encompassing primary and associative areas [e.g., anterior cingulate and anterior insula cortices (Ingvar, 1999; Boly et al., 2008; Shackman et al., 2011; Chatelle and Thibaut, 2014)] together with their preserved functional connectivity, have been observed in MCS patients, suggesting the possibility of integrated conscious processing of the pain. Nevertheless, these techniques are generally very expensive, complex, and time-consuming.

Alternative methods, based on the analysis of the Autonomic Nervous System (ANS), such as probing physiological signals of peripheral organs like the heart (Riganello and Sannita, 2009; Ryan et al., 2011; Riganello et al., 2012; Koenig et al., 2014) and skin conductance (Storm, 2008) have been proposed to overcome these issues.

By means of the GSR and HRV analysis, it is possible to observe the autonomic response to the nociceptive stimulation.

HRV is defined as the fluctuation in the time intervals between adjacent heartbeat and reflects at any moment the complex interactions with the ANS. It mirrors to a substantial extent the cardiorespiratory control system and is regarded as a reliable index of the sympathetic/parasympathetic functional interplay (Thayer, 2007; Riganello et al., 2012; Mather and Thayer, 2018). As the sequence of heartbeats is non-linear, the HRV is better described by the mathematical chaos as the HRV entropy analysis. Reduced HRV entropy suggests a less complex autonomic response to noxious stimuli in UWS patients (Riganello et al., 2018a; Tobaldini et al., 2018) and discriminates them from MCS patients (Riganello et al., 2018b).

Separately, the GSR measures the conductance of the skin that is related to the autonomic innervation of the sweat glands and reflects the activity of the sympathetic nervous system (Critchley, 2002).

Several studies highlight the potential of measuring the GSR following auditory (Hildebrandt et al., 1998; Keller et al., 2007; Dolce et al., 2008) or nociceptive stimuli in DOC patients (Venturella, 2018). Notwithstanding, these measures were not used in a systematic way to explore the pain perception in these patients.

Calabrò et al. (2017) combining EEG and HRV in an LEP study found preserved cortical activation and lower HRV in all MCS and in two UWS patients. Pain-related stimulation was also associated with a delta parietal response, lower left frontal activation, and increased GSR and heart rate (Venturella, 2018).

These measures do not require the person's collaboration or behavioral feedback, and have been proposed as a reliable indicator of nociceptive pain processing in different studies using hypnosis or analgesia in healthy individuals (Rainville et al., 1999; Jeanne et al., 2009).

In our study, we explored the possibility to observe the autonomic response related to a Trace Conditioning learning experiment, by means of a nociceptive stimulus in UWS patients, and if such learning may have a significance in the recovery of the consciousness.

Classical conditioning works by an associative process beginning with the presentation of a contingency between Conditioned Stimulus (CS) and Unconditioned Stimulus (US), in which a neutral (conditioned) stimulus (e.g., a tone) acquires a motivational feature after being paired with another, biologically evocative (unconditioned), stimulus (e.g., nociceptive stimulus) eliciting an unconditioned response (UR). In this way, the CS elicits a Conditioned Response (CR), despite the absence of the US (Çevik, 2014; Eelen, 2018).

Further, if two stimuli are presented together, only one will acquire the function of signal. Pavlov (1927) defined the difference between the stimuli as “saliency,” and Kelley and Michela (1980) considered it as the attribution of “an effect to the most salient cause in the perceptual field at the time the effect is observed.” Some interpretations suggested the idea that an effect referred to the first cause coming to mind or providing sufficient justifications. It seems possible that the saliency effect is mediated by the superior memory for the salient cause (Kelley and Michela, 1980).

The classical conditioning is an example of non-declarative or unconscious memory (hippocampus-independent), expressed by performance and without access to conscious memory (hippocampus-dependent), or awareness that memory is used (Clark, 1998).

The trace conditioning is a different version of classical conditioning, characterized by a short time interval between the conclusion of the CS and the presentation of the US (Pavlov, 1927), and is considered an adequate method to assess the consciousness presence without a verbal report (Bekinschtein et al., 2009).

The capability to form an internal representation of environmental contingency, in a symbolic or propositional way, makes it conscious. However, there is no clear agreement about the contingency awareness –i.e., the knowledge that a specific CS predicts a specific US– that is considered necessary but not sufficient by some reviewers and both necessary and sufficient by others (Lovibond and Shanks, 2002).

The relation between trace conditioning and conscious access to the CS or UC is debated. As reported, both comatose patients (Juan et al., 2016) and individuals during deep sleep (Arzi et al., 2012; Züst et al., 2019) were shown to be able to perform associative learning in the absence of consciousness.

The trace conditioning implies the involvement of the neocortex and hippocampus to represent and retain, respectively, the relationship between CS and US. Furthermore, the cerebellum warrants the performance of the Conditioned Response (CR) (Clark, 1998), i.e., the learned response to the previously neutral stimulus.

Bekinschtein et al. (2009) in a trace conditioning study on Disorders of Consciousness (DOC) patients, through the association of a tone with an air puff to the cornea to elicit an eye blink, reported that the patients might have a partially preserved conscious process.

In this study by the use of GSR and HRV entropy, for the first time, we wanted to observe the behavior of UWS patients during the administration of a noxious stimulus in a frame of trace conditioning, in order to discover an eventual consciousness activity. We hypothesized to find: (1) a higher GSR magnitude for the CR, (2) the extinction of CR, (3) the CR in UWS patients as a possible better prognosis, (4) higher values of HRV entropy in healthy controls (HC) if compared to the patients, and (5) higher values of HRV entropy in patients with CR if compared to the patients without CR.

## Methods

### Participants

We recruited 13 HC (7 females, mean age 34 ± 11, 6 males, mean age 35 ± 7) and 37 UWS patients at 31 ± 9 days from the acute event, of which seven were excluded because of signal artifacts, due to the movements during the recording. Of the 30 selected patients (Table 1), 13 were females (5 Hemorrhagic, 6 Traumatic, 2 Anoxic, mean age 44 ± 17, score range CRS-R [3–6], score range NCS [1–5]) and 17 males (5 Hemorrhagic, 9 Traumatic, 3 Anoxic, mean age 50 ± 20, score range CRS-R [2–6], score range NCS [1–5]). The enrolled patients were hospitalized in a special rehabilitation unit for UWS patients at the S. Anna Institute of Crotone (Italy). The inclusion criteria were: (1) age more than 16, (2) no administration of neuromuscular blockers or sedation within 24 h of enrolment, (3) eyes opening (indicating wakefulness and rest cycles), (4) diagnosis of UWS, based on behavioral assessments by way of CRS-R (Giacino et al., 2004), (5) stable clinical condition, and (6) time of recruiting no more than 30 days from the injury. Exclusion criteria were: (1) documented history of prior brain injury; (2) functional disability resulting from premorbid developmental, psychiatric, or neurologic illness; (3) upper and lower limb contusions, fractures, or flaccid paralysis; (4) neurological or psychiatric disease; and (5) administration of pharmacological drugs interacting with the level of consciousness.

**Table 1:** Demographic information of groups, test results and CRS-R/NCS assessments of UWS patients.

					**Protocol result**	**CRS-R/NCS**				
**Patient**	**Diagnosis**	**Age**	**Etiology**	**Time from event (days)**	**CR response**	**Week I**	**Week II**	**Week III**	**Week IV**	**Final diagnosis**
1	UWS	**50–55**	**HEM**	**27**	**1**	**4/3**	**4/3**	**8/4**	**8/4**	**MCS**
2		**60–65**	**HEM**	**39**	**1**	**6/5**	**7/7**	**9/6**	**11/7**	**MCS**
3		**16–20**	**TBI**	**28**	**1**	**4/1**	**4/1**	**7/3**	**8/3**	**MCS**
4		**66–70**	**TBI**	**25**	**1**	**6/5**	**6/5**	**10/8**	**12/7**	**MCS**
5		**60–65**	**TBI**	**26**	**1**	**4/3**	**5/3**	**9/5**	**8/5**	**MCS**
6		**30–35**	**TBI**	**21**	**1**	**5/2**	**5/2**	**8/4**	**10/5**	**MCS**
7		**66–70**	**TBI**	**39**	**1**	**4/4**	**8/3**	**8/5**	**8/5**	**MCS**
8		**16–20**	**TBI**	**25**	**1**	**4/2**	**8/3**	**14/5**	**14/7**	**MCS**
9		**36–40**	**ANOX**	**21**	**1**	**4/3**	**6/2**	**4/3**	**6/4**	**UWS**
10		66–70	HEM	23	0	4/4	7/3	4/3	7/5	UWS
11		40–45	HEM	55	0	4/1	4/1	4/1	4/1	UWS
12		46–50	HEM	32	0	4/1	3/4	3/3	5/4	UWS
13		56–60	HEM	24	0	5/3	4/3	5/3	5/3	UWS
14		56–60	HEM	35	0	4/3	4/3	4/3	6/3	UWS*
15		56–60	HEM	39	0	3/1	5/3	5/3	6/3	UWS
16		66–70	HEM	58	0	5/3	5/3	6/3	6/3	UWS
17		60–65	HEM	34	0	5/3	6/3	5/3	4/4	UWS
18		56–60	TBI	30	0	5/1	5/4	4/3	5/4	UWS
19		46–50	TBI	23	0	2/1	7/5	5/3	5/5	UWS
20		60–65	TBI	33	0	6/3	6/3	6/3	6/3	UWS
21		56–60	TBI	36	0	5/2	5/2	5/2	7/5	UWS
22		20–25	TBI	27	0	5/2	6/2	6/4	6/4	UWS
23		20–25	TBI	27	0	4/3	6/4	6/3	5/3	UWS
24		26–30	TBI	28	0	6/3	5/4	5/4	7/4	UWS
25		26–30	TBI	23	0	6/5	6/5	7/6	7/5	UWS
26		20–25	TBI	21	0	5/3	6/3	7/4	7/4	UWS*
27		66–70	ANOX	25	0	5/4	6/4	6/5	7/7	UWS
28		60–65	ANOX	23	0	5/1	5/2	4/2	4/2	UWS
29		26–30	ANOX	31	0	3/3	3/3	6/3	6/3	UWS
30		40–45	ANOX	37	0	4/1	5/2	6/2	5/3	UWS
1	HC	50–55								
2		20–25								
3		20–25								
4		30–35								
5		36–40								
6		26–30								
7		36–40								
8		20–25								
9		36–40								
10		36–40								
11		40–45								
12		26–30								
13		40–45								

MCS, Minimally Conscious State; UWS, Unresponsive Wakefulness Syndrome; UWS*, patients without CR that change the level of consciousness after 6 months from the onset; HEM, Hemorrhagic; TBI, Traumatic Brain Injury; ANOX, Anoxic; CR, Conditional Response (0 = absent; 1 = present); CRS-R, Coma Recovery Scale–Revised; NCS, Nociception Coma Scale; Week (I-IV), Successive weeks during which the patients were assessed by CRS-R and NCS. In bold patients with Trace Conditioning.

All recruited patients were evaluated by acoustic evocated potential to exclude any sensorial acoustic impairment.

The study was approved by the Ethics Committee and written informed consent was obtained by the HC and the patients' legal representative.

### Protocol

The patients were enrolled within 10 days from the hospitalization and evaluated 1 week before the start of the protocol by means of CRS-R, in order to verify the UWS condition, and by NCS to select the best responsive limb to the nociception stimulation.

The CRS-R consists of 23 hierarchically arranged items and comprises six subscales addressing arousal, auditory, visual, motor, oromotor/verbal, and communication functions. The NCS is structured in a similar way and consists of 16 hierarchically arranged items and comprises four subscales: visual, motor, verbal, and facial expression.

The lowest item on each subscale represents reflexive activity while the highest item represents cognitively-mediated behaviors.

The GSR and ECG were recorded by Nexus 10 (www.mindmedia.com). The GSR signal was acquired by two AgCl ring finger electrodes positioned on the index and medium fingers, with a 24-bit resolution able to register changes of <0.0001 microsiemens, at a sample rate of 32 Hz. The ECG was recorded by adhesive electrodes positioned on the chest of the patients at a sample rate of 128 Hz.

Two different stimuli were administered: (1) a musical stimulus (MUS) and (2) a noxious stimulus (NOC).

**Figure 1:**
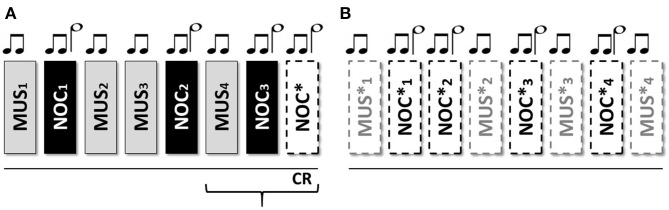
Sequence of the stimuli: two short grave notes anticipate the musical stimulus; two short grave notes and one long acute note at an interval of 5th anticipate the noxious stimulus. From Sequence **(A)** 1 to 7 the stimuli were associated to the tones; the eighth without stimulus administration to verify the CR; Sequence **(B)** from 9 to 16 only tones for the extinction of the stimulus.

Each stimulus was associated with a specific tone listened to before the administration. Different tones were associated with musical and noxious stimuli. We administered three nociceptive stimuli (none were administered after the fourth tone) to verify the presence of conditional learning (Figure 1). As in the fear conditioning (Hermans et al., 2006), it has been sufficient to use a limited amount of noxious stimuli in order to verify the occurrence of learnin‘g. The associative model was previously tested on voluntary HC.

**Figure 2:**
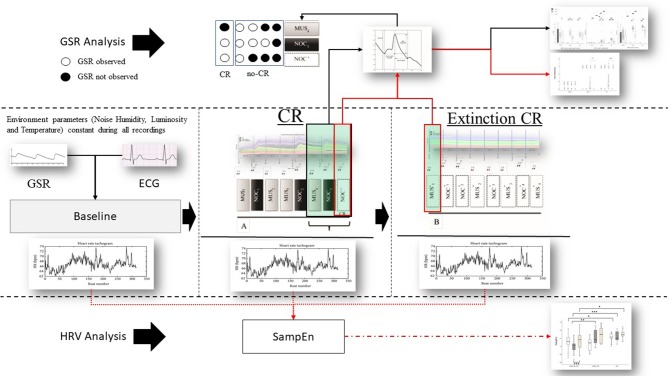
Protocol and data analysis: in the central line of the figure (between dashed lines) the entire sequence of the protocol (baseline, sequence A to test the conditional response (CR), and sequence B to test the extinction of the CR). From each sequence Galvanic Skin Response (GSR) and Electrocardiogram (ECG) were extracted. From the ECG the Inter-Beats Interval (distance between peak to peak of RR ECG signal) was extracted (Bottom the figure—HRV analysis) and Sample Entropy (SampEn) analyzed, then compared between and within groups. GSR was observed in baseline to exclude artifact movements. GRS analysis (above) was performed considering peak magnitude, time to reach the peak, and decay of the GSR signal in the last three phases of the sequence A (black box and black line MUS4, NOC3, and NOC*). It was considered the CR only if the GSR was present in NOC3 and NOC* (circle white filled). Finally, CR's (red box) extinction was observed in the sequence B. Similarly, for the SampEn, the GSR components (magnitude, time to reach the peak, and decay) were compared between and within groups (black line) and the extinction was compared for wave peak magnitude (red line).

The scheme consists of 16 stimuli (organized in 2 consecutive sequences A and B) administered, in one session, as follow: (A) MUS1 – NOC1 – MUS2 – MUS3 – NOC2 – MUS4 – NOC3 – NOC^*^ and (B) MUS1^*^, NOC1^*^, NOC2^*^, MUS2^*^, NOC3^*^, MUS3^*^, NOC4^*^, MUS4^*^, where the (^*^) indicates the only tones administration (Figure 2). In order to confirm the efficacy of the protocol and verify the presence of the association, the scheme was administered to the HC group.

Because of the conductance level of GSR (the start increases rapidly and decreases until to the baseline in an asymptotic way, following a slow exponential decay), the interval between consecutive stimuli was set on 25 s, to avoid overlapping with any of the GSR signals (Dawson et al., 2007; Breska et al., 2011).

**Figure 3:**
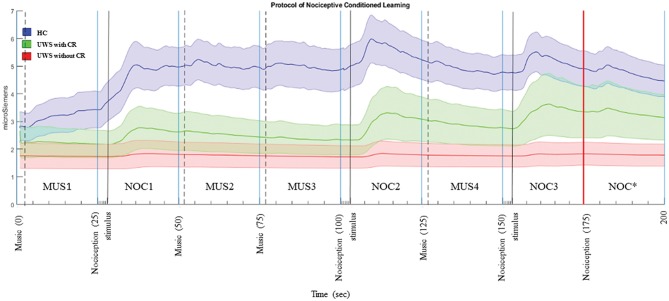
Phasic components of GSR in the sequence A. The bold line is the mean of the GSR signal, and the filled area is the standard error. In blue the HC, green UWS patients with CR, and red UWS patients without CR. Vertical black continuous lines are the start of nociception stimulus, dashed black lines are the start of musical stimulus, vertical red line is the start of CR. MUS4, NOC3, and NOC* were considered in the statistical analysis to verify the conditional learning.

Each stimulus was preceded by two different tones: (1) two short grave notes for the musical stimulus and (2) two short grave notes and one high long note at an interval of 5th (i.e., do-sol) for the noxious stimulus. The interval inter-stimuli was 1 s. The administered musical stimulus was the first movement of the Beethoven's Symphony no. 6, played by two speakers positioned behind the subject, and fading out during the last 5 s (Figure 3). The noxious stimuli were interposed with musical stimuli in order to reduce the arousal level (Khalfa et al., 2002; Lee, 2003) and to avoid a prolonged silence period (Blain et al., 2010; Lui and Grunberg, 2017), interfering with the GSR signal.

The noxious stimulus was administered by a Newton-meter (Force Dial, FDN 200 model; Connecticut, USA; www.wagnerinstruments.com) which allows the examiner to gauge the amount of pressure applied to the patient (Schnakers et al., 2010) following the procedure described by the NCS (i.e., pressure on the nail bed for a maximum of 5 s, or interrupted at the first behavioral response of the subject).

The second part of the scheme (sequence B) aimed at observing the extinction of the CR (Figure 2).

During the recordings, HC were sitting comfortably on a chair with eyes closed and relaxed hands positioned on a small table, while the patients were sitting in a wheel-chair or in the bed. The protocol of stimulation was preceded by a 5-min baseline, in absence of any transient noise in order to reach a relaxed condition and a stable GSR signal.

All the recordings occurred in a condition of constant luminosity, humidity, temperature (24°C), in the absence of transient noise, and avoiding any influence from nursing and feeding or rehabilitative programmes. Scales administrations and the protocol of the trace conditioning were planned between 09:30 a.m. and 11:00 a.m., in order to obtain the best possible response and avoid differences due to the different moments of the day (Candelieri et al., 2011; Cortese et al., 2014).

### Data Extraction

A week before the protocol administration, the consciousness level of the patients and the response to noxious stimulus were assessed by means of CRS-R and NCS, respectively. Further, the assessments were repeated during the protocol administration and for 3 consecutive weeks.

The phasic wave signal of the GSR was extracted by Ledalab (Benedek, 2016). The last three stimuli of the sequence A (i.e., MUS4, NOC3, and NOC^*^) were considered to verify the CR.

The GSR and ECG signals were previously controlled, in order to avoid the presence of movement artifacts and missing data, respectively.

**Figure 4:**
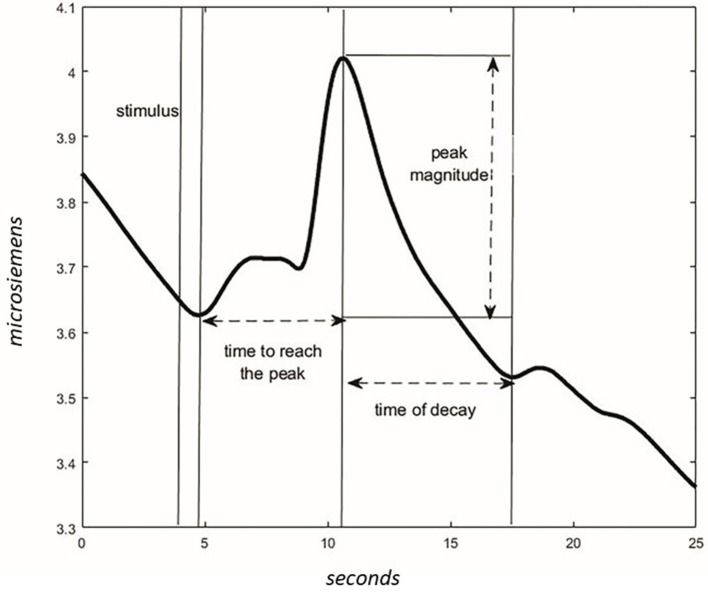
Characteristics of the wave in the GSR signal. After the stimulus, the time to reach the peak of max magnitude is calculated. The magnitude is different between values of peak and values at the start of the stimulus. The time of the wave's decay is the phase of decreasing after reaching the peak.

For each phase, the wave peak magnitude, wave's decay time, and time to reach the peak following the acoustic stimulus were extracted (Figure 4). The presence of CR was considered significant if the peak magnitude -within 10 s from stimulus administration- was higher in NOC^*^ than MUS 4 and higher in NOC3 than in NOC^*^.

The tachogram (the series of consecutive intervals between heartbeats) was extracted from the electrocardiogram, and the Sample Entropy (SampEn) was analyzed for the baseline, sequence A, and sequence B using the HRV Advanced Analysis software (Tarvainen et al., 2014).

$$\begin{array}{l} S_{E}\left(m,r,N\right)=-\text{ln}\frac{{ø}^{m+1}\left(r\right)}{{ø}^{m}\left(r\right)} \end{array}$$

Equation 1: *SE: Sample Entropy; m*: distance between time series points to be compared; *r*: radius of similarity; *N*: length of the time series; ϕ: probability that points *m* distance apart would be within the distance *r*.

Sample entropy (Equation 1) has been suggested to be independent from data length and shows consistency over broad ranges of possible data sequence length to be compared (m), tolerance (r), and total RR (interval from the R peaks of ECG QRS complex) interval data length (N) (Richman and Moorman, 2000; Yentes et al., 2013). The parameters m and r were set to 2 and 0.15, respectively, as used in previous studies (Costa et al., 2005; Costa and Goldberger, 2015).

### Statistical Analysis

Because of the dimension of our sample size and the violation of the homogeneity of variance, non-parametric tests were used. The exact test was used because it would be more accurate in the case of a small sample, or when the tables are sparse or unbalanced. Further, to extract the model useful to detect the presence/absence of CR by GSR, and test it (by the 10-fold-cross-validation), the Machine Learning method was used.

In order to verify the CR in NOC^*^, peak magnitude, time to reach the peak, and time of wave's decay were compared using Wilcoxon's test in MUS4, NOC3, and NOC^*^.

The effect size r was calculated as absolute value of Z/√(N) where Z is the Z-statistic (Rosenthal, 1991; Fritz et al., 2012) of the statistical test and N is the total number of subjects. The effect size results were considered: *r* < 0.1 not significant; 0.1 ≤ *r* < 0.3 low; 0.3 ≥ *r* < 0.5 medium; *r* ≥ 0.5 high (Fritz et al., 2012).

The UWS patients' group was divided into two subgroups: (1) UWS0: patients without CR at NOC^*^; (2) UWS1: patients with CR to NOC^*^, then MUS4, NOC3, and NOC^*^ were compared as in HC for wave peak magnitude, time to reach the peak, and time of wave's decay using Wilcoxon's test.

The same parameters were used to compare HC, UWS0, and UWS1 groups between them using Mann-Whitney test.

To evaluate the extinction of the CR, MUS1^*^, and NOC1^*^ of the sequence B were compared with NOC^*^ and MUS4 of the sequence A for wave peak magnitude using Wilcoxon's test. The *p*-value of the test was set to *p* < 0.005 for multiple comparisons.

The different scores of CRS-R and NCS in the 4 weeks were compared using Wilcoxon's test.

The results of the model (number of UWS patients with CR that changed level of consciousness) were evaluated for sensitivity (rate of patients with change in level of consciousness correctly classified); specificity (rate of patients without change in the level of consciousness correctly classified); precision (rate of correct prediction in the change of level of consciousness), false positive and negative rates in the classification of change of the level of consciousness, and accuracy (predicted conditions of change and no change in the level of consciousness).

HC, UWS0, and UWS1 groups were compared among them for SampEn in baseline, sequence A, and sequence B using Mann-Whitney's test. Moreover, baseline vs. sequence A, and sequence A vs. sequence B were compared in all groups using Wilcoxon's test.

We used WEKA (Waikato Environment for Knowledge Analysis), an open source toolbox for machine learning analysis, and the One-R classifier to generate the simplest rule for discriminating the presence/absence of the CR, by means of SampEn or GSR parameters recorded in MUS4, NOC3, and NOC^*^. One-R (Holte, 1993) is a fast and very simple algorithm deriving a one-level decision tree. It operates by generating a separate rule for each individual attribute of the dataset based on error rate. To generate the rule, each attribute is discretized into bins calculating the percentage that each class (presence/absence of the CR) appears within each bin. Finally, the rule for the final decision tree is chosen by selecting the attribute with minimum error to perform the classification.

The whole data set was used to generate the model (training test), then the 10-fold cross-validation test was used to assess the performance of the model in generalization. The results of the training and 10-fold cross-validation tests were calculated by several metrics: sensitivity (rate of conditional learning correctly classified), specificity (rate of no conditional learning correctly classified), false positive and negative rates of conditional and no conditional learning classification, accuracy (conditional learning and no conditional learning predicted conditions), precision (rate of correct prediction in the assessment of conditional learning), and F1-score [a measure of the test's accuracy that takes in consideration the harmonic mean of sensitivity and its precision—ranging values: (0—worst precision and sensitivity: 1–perfect precision and sensitivity)].

## Results

**Figure 5:**
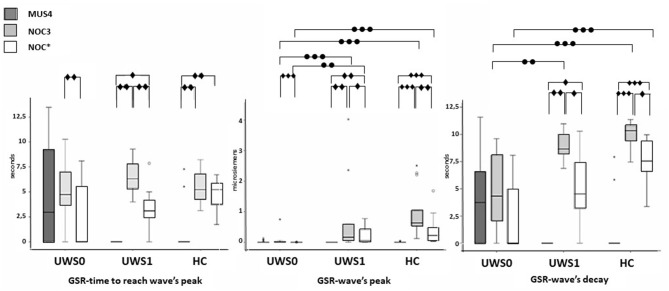
Sequence A: boxplot of the GSR waves components. Time to reach the peak (left), wave's magnitude (center) and wave's decay (right) of each group were compared among them in MUS4, NOC3, and NOC* (diamond markers), and the groups (HC, UWS1, and UWS0) were compared among them for each session (point markers). The box represents the first and third quartile, the whiskers are the 1.5 interquartile range, the black lines are the medians, and points are outliers. Significant statistical difference: ^∙^*p* = [0.002–0.003]; ^∙∙^*p* = 0.001; ^∙∙∙^*p* < 0.0001; circle: Statistical difference between groups .

All subjects of the HC group showed a CR in the NOC^*^. A Wilcoxon's test for the GSR magnitude of CR was higher compared to MUS4 (*Z* = −3.180, *p* < 0.0001, *r* = 0.62) and lower compared to NOC3 (*Z* = −3.110, *p* = 0.001, *r* = 0.61). No significant difference was found in the time to reach the peak between NOC3 and NOC^*^ (*Z* = −2.900, *p* = 0.001, *r* = 0.61), while the time of wave's decay was higher in NOC3 (*Z* = −2.621, *p* = 0.003, *r* = 0.44) (Figure 5, Table 2).

**Table 2:** Statistic results for GSR in MUS4, NOC3, and NOC^*^ and for SampEn in Baseline, sequence A and sequence B.

	**Galvanic Skin Response**								
	**Time to reach weave's peak**			**Wave's peak magnitude**			**Wave's decay**		
	**Music**	**Nociception**	**Test**	**Music**	**Nociception**	**Test**	**Music**	**Nociception**	**test**
UWS0 vs. UWS1	ns	ns	ns	ns	*Z* = −3.345, *p* = •••, *r* = **H**	*Z* = −3.614, *p* = •••, *r* = **H**	ns	*Z* = −2.949, *p* = ••, *r* = **H**	ns
UWS1 vs. HC	ns	ns	ns	ns	ns	ns	ns	ns	ns
UWS0 vs. HC	ns	ns	ns	ns	*Z* = −4.533 *p* = •••, *r* = **H**	*Z* = −4.880, *p* = •••, *r* = **H**	ns	*Z* = −4.312 *p* = •••, *r* = **H**	*Z* = −4.205, *p* = •••, *r* = **H**
	**UWS0**			**UWS1**			**HC**		
	**Time to reach weave's peak**	**Wave's peak magnitude**	**Wave's decay**	**Time to reach weave's peak**	**Wave's peak magnitude**	**Wave'sdecay**	**Time to reach weave's peak**	**Wave's peak magnitude**	**Wave's decay**
Music vs. Nociception	ns	ns	ns	*Z* = −2.803, *p* = ••, *r* = **H**	*Z* = −2.803, *p* = ••, *r* = **H**	*Z* = −2.803, *p* = •• *r* = **H**	*Z* = −2.900, *p* = ••, *r* = **H**	*Z* = −3.180, *p* < •••, *r* = **H**	*Z* = −3,180, *p* = ••• *r* = **H**
Nociception vs. Test	*Z* = −2.864, *p* = ••, r = **M**	*Z* = −3.337, *p* = •••, *r* = **H**	ns	*Z* = −2.803, *p* = ••, *r* = **H**	*Z* = −2.701, *p* = •, *r* = **H**	*Z* = −2.666, *p* = •, *r* = **H**	ns	*Z* = −3.110, *p* = ••, *r* = **H**	*Z* = −2.621, *p* = •, *r* = **M**
Music vs. Test	ns	ns	ns	*Z* = −2.666, *p* = •, *r* = **H**	*Z* = −2.666 *p* = •, *r* = **H**	*Z* = −2.666, *p* = •, *r* = **H**	*Z* = −3.040 *p* = ••, *r* = **H**	*Z* = −3.180, *p* < •••, *r* = **H**	*Z* = −3,110, *p* = •••, *r* = **H**
	**Heart Rate Variability—Sample Entropy**								
	**UWS0 vs. UWS1**	**HC vs. UWS1**	**UWS0 vs. HC**			**Baseline vs. trace conditioning**		**Trace conditioning vs. extinction**	
Baseline	ns	ns	ns		UWS0	ns		*Z* = −2.527, *p* = •, *r* = **M**	
Trace Conditioning (Sequence A)	*Z* = −3.036, *p* = ••, *r* = **H**	ns	*Z* = −4.237, *p* = •••, *r* = **H**		UWS1	ns		ns	
Extinction (Sequence B)	ns	ns	*Z* = −2.726, *p* = •, *r* = **M**		HC	ns		ns	

*UWS0, Unresponsive Wakefulness syndrome patients with no Conditional Response; UWS1, Unresponsive Wakefulness syndrome patients with Conditional Response*.

*p-value: ••• p < 0.0001, r-value: M (effect size Medium 0.3 ≤ r < 0.5)*.

*p-value: ••p = 0.001, r-values: H (effect size High r ≥ 0.5)*.

*p-value: 0.002 ≤ p ≤ 0.004*.

Of the 30 patients, nine (30%) showed a CR to the noxious stimulus (UWS1) with superimposable results at Wilcoxon's test, with a higher GSR magnitude of CR compared to MUS4 (*Z* = −2.666 *p* = 0.002, *r* = 0.63) and lower compared to NOC3 (*Z* = −2.701, *p* = 0.002, *r* = 0.64), and time to reach the peak shorter in NOC3 compared to NOC^*^ (*Z* = −2.803, *p* = 0.001, *r* = 0.66). In patients without CR (UWS0), significant differences were found between NOC3 and NOC^*^ for magnitude (*Z* = −3.337, *p* < 0.0001, *r* = 0.51) and time to reach the peak (*Z* = −2.864, *p* = 0.001, *r* = 0.44) (Figure 5, Table 2).

**Figure 6:**
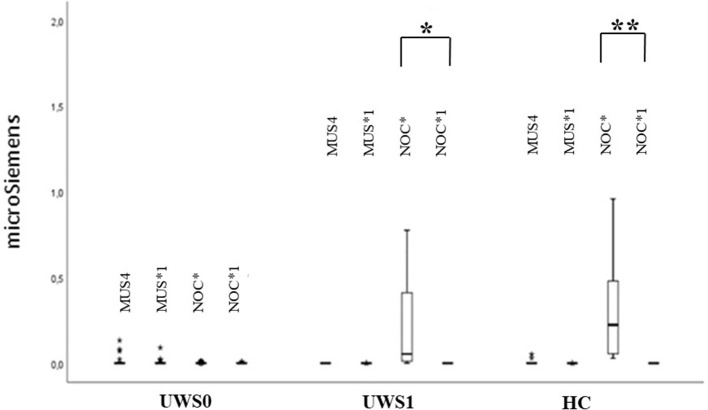
Sequence B: boxplot of GSR magnitude. MUS4 vs. MUS1* and NOC* vs. NOC1* were compared. Only in the HC and UWS1 groups a significant difference between NOC* and NOC1* was observed (***HC: *Z* = −3.180, *p* < 0.0001, *r* = 0.88; **UWS1: Z = −2.666; *p* = 0.002; *r* = 0.60). The box represents the first and third quartile, the whiskers are the 1.5 interquartile range, the black lines are the medians, and points are outliers.

The extinction of CR in the GSR signal was observed in HC (*Z* = −3.180, *p* < 0.0001, *r* = 0.88) and UWS1 (*Z* = −2.666, *p* = 0.002, *r* = 0.60) comparing NOC^*^ and NOC1^*^ (Figure 6).

No significant difference was found comparing UWS1 and HC for all wave's components of the GSR. Conversely, significant differences were found comparing UWS0 vs. UWS1 in NOC3 and NOC^*^ for peak magnitude (−3.614 ≤ *Z* ≤ −2.801; 0.0001 ≤ *p* ≤ 0.003; *r* = 0.66), and in NOC3 for wave's decay (*Z* = −2.949; *p* = 0.001). Comparing HC vs. UWS0 differences in NOC3 and NOC^*^ were found for peak magnitude and wave's decay (Z < −4.205; *p* < 0.001).

**Figure 7:**
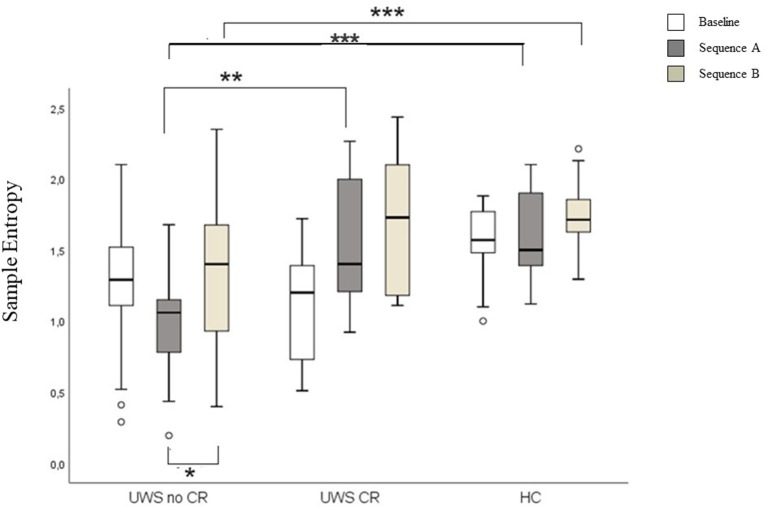
Boxplot of the SampEn. In the figure: baseline (white), sequence A (dark gray) and sequence B (light gray). Significant statistical difference: *p = [0.003–0.005]; **p = [0.001]; ***p < 0.0001.

Higher SampEn was found comparing UWS1 vs. UWS0 in the sequence A, and in HC vs. UWS0 sequence A and sequence B (Mann-Whitney's test: *Z* ≥ −2.726; *p* ≥ 0.005; *r* ≥ 0.47). Significant differences were found for SampEn in UWS0 comparing sequence A vs. sequence B (Wilcoxon' test: *Z* = −2.573, *p* = 0.004; *r* = 0.40) (Figure 7, Table 2).

**Figure 8:**
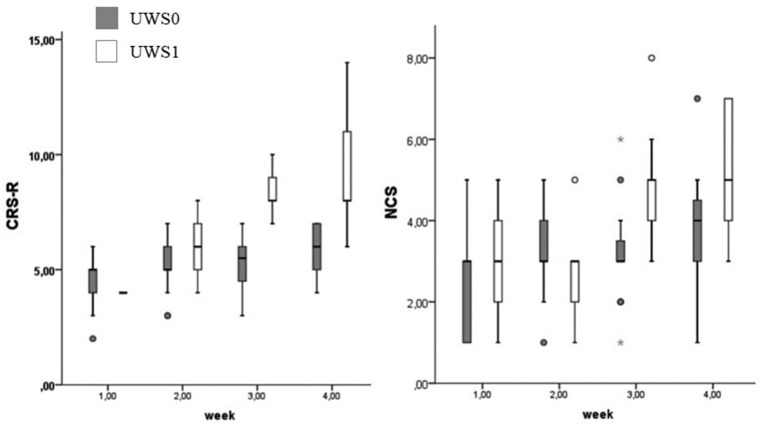
Boxplot of the CRS-R and NCS. UWS0 (dark gray) and UWS1 (white) groups are compared for CRS-R and NCS. The box represents the first and third quartile, the whiskers are the 1.5 interquartile range, the black lines are the medians, and points are outliers. The statistical difference between groups is significant at the 3rd week (CRS-R: Z = −3.512; *p* < 0.0001 – NCS: *Z* = −2.964; *p* = 0.001) and 4th week (CRS-R: *Z* = −3.566; *p* < 0.0001 – NCS: *Z* = −2.214; *p* = 0.015).

At Wilcoxon's test UWS0 and UWS1 were different for CRS-R and NCS in the third (CRS-R: *Z* = −3.512; *p* < 0.0001 – NCS: *Z* = −2.964; *p* = 0.001) and fourth week (CRS-R: *Z* = −3.566; *p* < 0.0001 – NCS: *Z* = −2.214; *p* = 0.015) (Figure 8). In this range of time, eight of the nine patients (88.9%) with a positive conditional learning to the noxious stimulus showed behaviors according to MCS (Table 1). Only two of the UWS0 patients showed an evolution of the level of consciousness, but only after 6 months from the onset.

The CR observed by the protocol showed a high prognostic power to predict the change of the level of consciousness, with a sensitivity of 100%, specificity of 95%, accuracy of 97%, precision of 90%, and a false positive and negative rate of 5 and 0%, respectively (Table 3).

**Table 3:** Results of the prognostic power the CR observed by the protocol, and results of the One-R classifier in the correct classification of the presence/absence of the CR.

		**Correct Classification of presence/absence of the CR based on One-R**						
		Parameter: Peak magnitude NOC* **Rule: magnitude <0.05 → no CR**			**Parameter: Peak magnitude NOC3Rule: magnitude <0.105 → no CR**		**Parameter: SampEn Rule: SampEn <1.245 → no CR Sequence A**	
**Presence of the nociceptive conditional learning and change in the level of consciousness in UWS patients**			**Training test**	**10-fold cross-validation test**	**Training test**	**10-fold cross-validation test**	**Training test**	**10-fold cross-validation test**
Sensitivity (%)	100	Sensitivity (%)	**96**	**95**	95	86	86	71
Specificity (%)	95	Specificity (%)	**95**	**95**	95	85	86	67
Accuracy (%)	97	Accuracy (%)	**95**	**91**	91	86	84	72
Precision (%)	90	Precision (%)	**95**	**86**	86	86	82	77
False Positive Rate (%)	5	False Positive Rate (%)	**5**	**13**	13	14	18	26
False Negative Rate (%)	0	False Negative Rate (%)	**5**	**5**	5	14	14	29
		F1 score [0:1]	**0.95**	**0.94**	0.9	0.86	0.84	0.74

The best performance in bold (Peak magnitude NOC*). Additionally, the results with the parameters Peak magnitude NOC3 and SampEn.

By means of One-R classifier of WEKA, the peak magnitude of the GSR in NOC^*^ was selected for the best correct classification of the CR and groups. The classifier showed a high performance in the training test to differentiate presence/absence of CR, with a sensitivity, specificity, accuracy, and precision between 95 and 96%, and values of F1 score equal to 0.95. The correct classification was also high at the 10-fold cross-validation test with a sensitivity, specificity, accuracy, and precision between 86 and 95%, F1 score 0.93 (Table 3). Additionally, the correct classification by means of the peak magnitude NOC3 and of the SampEn in the sequence A was also tested (Table 3).

## Discussion

As of today, the UWS condition represents as an ethically troublesome condition which is hard to fully understand. The principal methods for the diagnosis of the patients with severe DOC are based on behavioral scales, such as CRS-R. About the evaluation of the pain perception, the NCS represents a valid instrument to differentiate a generalized response from a specific response to the nociceptive stimulus (Schnakers et al., 2010; Riganello et al., 2014; Chatelle and Laureys, 2015). However, there is a higher probability of a diagnostic error when the patient does not present any cognitive output. In fact, the misdiagnosis of DOC patients is again around 30% (Andrews et al., 1996; Bosco et al., 2010; van Erp et al., 2015).

EEG and neuroimaging tools and studies help the clinicians in the diagnosis, prognosis, and decision making in DOC patients (Gantner et al., 2012; Fernández-Espejo and Owen, 2013; Di Perri et al., 2014; Demertzi et al., 2015), and provide evidence for the correlation between cortical activation and response to the noxious stimulus (Kassubek et al., 2003; Markl et al., 2013; Tommaso et al., 2013; Chatelle and Thibaut, 2014; Naro et al., 2016). However, these approaches are not always possible or practicable in DOC patients with clinical conditions because, as for the fMRI, they are expensive, complex, and time-consuming.

The association of the GSR to the nociceptive assessment, and in particular the conditional learning, can represent a complementary instrument to increase the suitability of assessment of patients with DOC.

By means of the HRV entropy analysis, and specifically the Complexity Index, a recent study showed that the Central Autonomic Network (CAN) [a brain-heart integrated model (Riganello, 2016) in which neural structures are involved in cognitive, affective, and autonomic regulations] modulates a different response to the noxious stimulus, among HC, MCS, and UWS patients. The clear decreasing modulation in UWS patients supported the idea of a correlation with the reduced level of consciousness (Riganello et al., 2018a).

Again, a resting-state fMRI study showed a correlation between HRV complexity and the level of consciousness, in particular with the Fronto-Insular cortex, Superior Frontal Gyrus, Paracingulate cortex, Insular cortex, Dorso-Lateral Prefrontal Cortex, Superior Parietal Lobule, and Superior Temporal Gyrus (Riganello et al., 2018b).

In this study, we analyzed the CR to the nociceptive stimulus in UWS patients in the early phase of hospitalization, and its prognostic valence. The most evident result was the different responses obtained in terms of the wave's magnitude. The HC group showed a greater GSR at NOC3 when compared to the UWS patients. Moreover, although CRS-R and NCS were not significantly different, the UWS1 group had a higher GSR at NOC3 if compared to UWS0. No significant difference between the HC and UWS1 groups was found in the SampEn, while it was higher in UWS1 compared to UWS0. Further, this last group showed lower values of the SampEn in the sequence A (trace conditioning), when compared to sequence B (extinction of CR), confirming the results reported in previous studies and suggesting a less complex autonomic response to noxious stimuli in UWS patients without CR.

The UWS1 patients showed behaviors overlapping with MCS within the following 4 weeks. Only one anoxic patient did not show any change, probably due to a worsening of the clinical conditions.

Separately to the HRV that is correlated to both parasympathetic and sympathetic branches of the ANS, the GSR is correlated only to the sympathetic system and is technically simple to use.

The GSR signal depends on the change of the skin conductance in response to sweat secretion (Roy et al., 2012). The sweat glands are innervated by post-ganglionic sudomotor fibers that trigger their activity (Kennedy et al., 1994; Riedl et al., 1998). The skin conductance response corresponds to the burst of the sudomotor nerve, that is linearly related to the number of recruited sweat glands and to the amplitude of the GSR (Freedman et al., 1994; Dawson et al., 2007).

The GSR is influenced by several brain regions with distinct anatomical contributions in the control of skin conductance response. In the behavioral emotional response, the ventromedial prefrontal cortex is involved in the GSR anticipatory response and the amygdala is implicated in the response to the learned association between stimulus and reinforcement (Critchley, 2002). The Anterior Cingulate Cortex (ACC) plays a role in integrating autonomic bodily states with behaviors, with the anticipatory response in the risk context and with volitional modulation (Critchley, 2002; Critchley et al., 2003; Roy et al., 2012).

In the pain matrix, the ACC within the prefrontal cortex also plays the role of encoding affective–cognitive information (Medford and Critchley, 2010).

The presence of conditional learning to the nociceptive stimulus might indicate a subcortical-cortical and cortico-cortical preserved brain areas activation.

However, the amplitude of the GSR presents inter- and intra-individual variability (Baba et al., 1988; Arunodaya and Taly, 1995) and is influenced by several factors, such as ambient temperature (Yokota et al., 1959), skin temperature (Fujimori, 1956; Levy et al., 1992), stimulus strength (Yokota et al., 1959; Hoeldtke et al., 1992; Arunodaya and Taly, 1995), mental emotional status (Knezevic and Bajada, 1985), and arousing stimulus and habituation (Elie and Guiheneuc, 1990).

To control these potential sources of variability, the environment setting of stimulation was maintained to ensure a constant level for noise, temperature, light, and humidity, in order to link the signal of the patient's response to the stimulus. Moreover, the patients were stimulated in the morning to avoid possible differences in the response due to fluctuation of the consciousness level (Candelieri et al., 2011; Cortese et al., 2014).

The detection, by the GSR signal, to the CR implies a more complex level of functioning of the ANS, as found by the higher SampEn observed in the UWS1 group.

The machine learning model, by mean of the One-R classifier, confirmed the validity of results, with very high values of suitability in the training test as well as in the 10-fold cross validation test. The level of sensitivity, specificity, and accuracy of the protocol provided evidence for the potentiality to discover potential covert consciousness activity in an early period, not otherwise observable with the current behavioral scales.

In our study, the patients that changed the level of consciousness in MCS showed low values at CRS-R total scores, but a conditional learning to the nociceptive stimulus, regardless of the etiology. The difficulty for the examiner to assess and objectivate the residual cognitive function could be due to the inconsistent, minimal, and difficult output to be detected (Owen et al., 2007; Bayne et al., 2016).

Differently from the study of Bekinschtein et al. (2009) (where the conditional learning in UWS patients was evaluated by 140 trials, [70 tones paired with as air-puff and 70 unpaired tones] evidencing as they may have preserved conscious process), our study is based on a protocol consisting of two consecutive sequences (A and B), administered in one session and on the evaluation of the response to the nociceptive stimulus already assessed by NCS. Further, the possibility to observe in the early period of hospitalization the presence of conditional learning to the nociceptive stimulus could contribute to a more correct diagnosis and prognosis in DOC patients and help in the rehabilitative phase (de Tommaso et al., 2015; Chatelle et al., 2018).

Although some variables have been considered and controlled, others such as etiology and correlated damage of the Central Nervous System could alter the sensorial sensibility of the patients and then the generation of a normal GSR signal (Vetrugno et al., 2003).

The lack of the GSR baseline with only tones (preceding the entire protocol administration) may be a limitation of the study, however, no variations have been detected to the GSR signal during the sequence B (extinction phase). The observation of the SampEn in the three different moments of the protocol (baseline, sequence A, and sequence B) represents a point of strength. The possible variation of entropy due to age (HRV entropy decreases with the age) and gender (HRV entropy is higher in females) (Umetani et al., 1998; Corrales et al., 2012; Voss et al., 2012) suggests that more studies are needed. The results show that the GSR is a good tool picking up eight out of the ten patients, that ultimately evolve to MCS. This suggests that other markers might be needed to completely reduce actual misdiagnosis.

The accurate and reliable evaluation of the level of consciousness is important for a more effective rehabilitative project. In this frame, the evaluation of the CR to the nociceptive stimulus can represent a complementary and simple tool to add to the behavioral assessment and clinical consensus. It provides a simple way to observe a response and formulates a possible prognosis in patients that may have a preserved conscious process, but where the response is undetectable because of the impossibility to exhibit intentional movements or verbal responses.

## Data Availability Statement

All datasets generated for this study are included in the article/supplementary material.

## Ethics Statement

The studies involving human participants were reviewed and approved by REGIONE CALABRIA COMITATO ETICO SEZIONE AREA CENTRO. Written informed consent to participate in this study was provided by the participants' legal guardian/next of kin.

## Author Contributions

DC, FR, and FA conceived, planned, conducted the research, interpreted the results, and drafted the manuscript. FR performed the analysis. CS and SL supervised the manuscript. All authors provided critical feedback and helped shape the final manuscript.

### Conflict of Interest

The authors declare that the research was conducted in the absence of any commercial or financial relationships that could be construed as a potential conflict of interest.
